# Eosinophil-Derived Neurotoxin (EDN/RNase 2) and the Mouse Eosinophil-Associated RNases (mEars): Expanding Roles in Promoting Host Defense

**DOI:** 10.3390/ijms160715442

**Published:** 2015-07-08

**Authors:** Helene F. Rosenberg

**Affiliations:** Inflammation Immunobiology Section, National Institute of Allergy and Infectious Diseases, National Institutes of Health, Bethesda, MD 20892, USA; E-Mail: hrosenberg@niaid.nih.gov; Tel.: +1-301-402-1545; Fax: +1-301-480-8384

**Keywords:** inflammation, leukocyte, evolution, chemoattractant

## Abstract

The eosinophil-derived neurotoxin (EDN/RNase2) and its divergent orthologs, the mouse eosinophil-associated RNases (mEars), are prominent secretory proteins of eosinophilic leukocytes and are all members of the larger family of RNase A-type ribonucleases. While EDN has broad antiviral activity, targeting RNA viruses via mechanisms that may require enzymatic activity, more recent studies have elucidated how these RNases may generate host defense via roles in promoting leukocyte activation, maturation, and chemotaxis. This review provides an update on recent discoveries, and highlights the versatility of this family in promoting innate immunity.

## 1. Introduction

**Figure 1:**
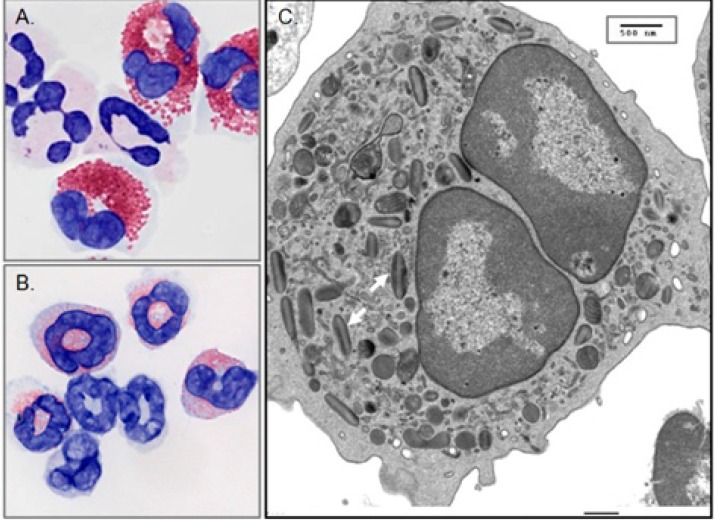
Human and mouse eosinophils. (**A**) Human eosinophils from peripheral blood exhibit characteristic bilobed nuclei and large red-stained cytoplasmic secretory granules; (**B**) Eosinophils isolated from the spleen of an interleukin-5 transgenic mouse. Cells in panels (**A**,**B**) were stained with the modified Giemsa protocol. Cells with multi-lobed nuclei and without large granules are neutrophils; original magnification, 100×; (**C**) Transmission electron micrograph of a mouse eosinophil. Two of the cytoplasmic secretory granules are indicated by white arrows just to the left of the lower lobe of the nucleus. The central core of the eosinophil specific granule contains the cationic major basic protein (MBP). The remaining major cationic proteins, including EDN, ECP and EPX, as well as cytokines, chemokines, growth factors and enzymes are localized in the peripheral portion of the granule; original magnification 6000×. Reprinted from [1] with permission from Nature Publishing Group.

The eosinophil-derived neurotoxin (EDN/RNase 2) is one of the four major secretory proteins found in the specific granules of the human eosinophilic leukocyte (Figure 1). EDN, and its more highly charged and cytotoxic paralog, the eosinophil cationic protein (ECP/RNase 3) are released from eosinophil granules when these cells are activated by cytokines and other proinflammatory mediators [1,2]. Although EDN expression has been detected in cells other than eosinophils, specifically monocytes and dendritic cells (see http://www.genecards.org) as well as in basophils and neutrophils, this protein is best known as an eosinophil constituent and is present in this cell in comparatively high concentration [3]. Recent interest in EDN has focused on its role as a specific biomarker in eosinophil-associated pathophysiologies, including asthma exacerbations [4,5,6,7], cow’s milk allergy [8], eosinophilic esophagitis [9], and vaccine-induced aberrant responses [10]. This review will focus on the antimicrobial properties of EDN and the mouse orthologs of the EDN/ECP gene pair; the unique antimicrobial features of ECP will be reviewed elsewhere in this Special Issue. Furthermore, there are several recent reviews that consider eosinophils and the specific granule proteins in greater detail [1,11,12,13,14,15].

## 2. Discovery, Isolation and Characterization of EDN

Gordon [16] was the first to detect EDN, albeit inadvertently, as a component of his studies related to the etiology of Hodgkin’s disease. Although Gordon believed that he was examining neurotoxic responses to a thermostable virus, Durack and colleagues [17] determined that eosinophils were the source of this activity. Durack and Gleich [18] ultimately purified an 18 kDa protein that reproduced “the Gordon phenomenon”, a syndrome of cerebellar dysfunction in association with loss of Purkinje cells in response to intrathecal injection, typically observed in experimental rabbits and guinea pigs. In a parallel effort, Peterson and Venge [19] purified the same granule protein, which they named eosinophil-protein X, or EPX. Both names can be found in the literature, although EDN will be used here so as to avoid confusion with the gene name for eosinophil peroxidase. EDN is also the same protein as liver RNase and also the same protein as non-secretory ribonuclease from human urine (RNase Us) [20]. As liver tissue is enriched with cells of the monocyte/macrophage lineage (and not eosinophils), this leukocyte lineage may be the source of liver (and urinary) EDN/RNase 2.

## 3. EDN Is Member of the RNase A Family of Secretory Ribonucleases

**Figure 2:**
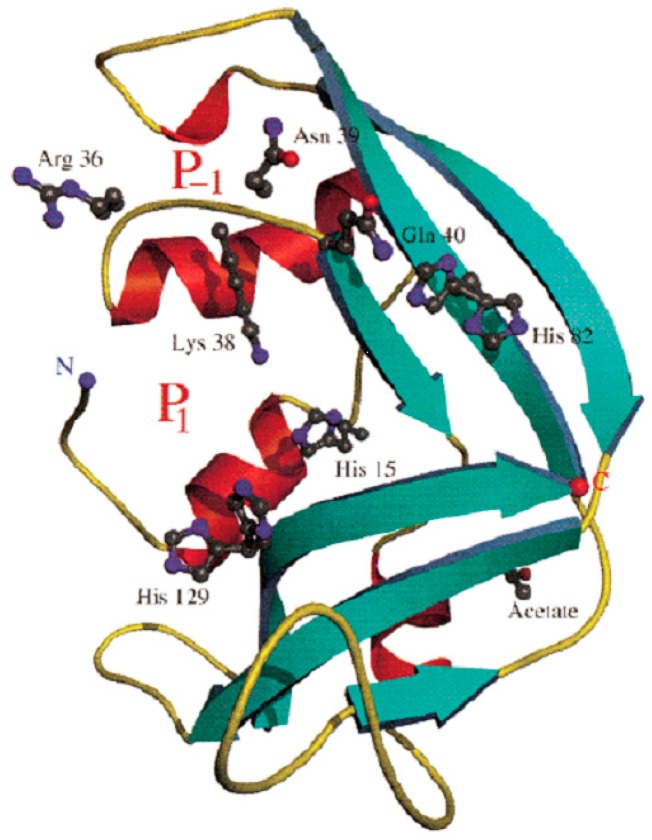
Ribbon diagram of the three-dimensional structure of human EDN. The relative positions of catalytic His 15, His 129, and Lys 38 are as shown, structure determined at 0.98 angstroms. Reprinted from [30] with permission from American Chemical Society.

In 1986, Gleich and colleagues [21] published the amino terminal sequence of EDN, which established homology between EDN and the eosinophil cationic protein, and between these two eosinophil proteins and human pancreatic ribonuclease. Shortly thereafter, Slifman and colleagues [22] found that EDN was an active ribonuclease, and was capable of generating soluble ribonucleotides from insoluble tRNA to an extent similar to that achieved by bovine pancreatic RNase A. The full nucleotide sequence of EDN was assembled from cDNAs identified in libraries generated from bone marrow RNA from an individual with eosinophil leukemia [23] and from cells of the HL-60 promyelocyte line [24]. The encoded amino acid sequences confirmed definitive homology to RNase A and to other members of the emerging RNase A gene family, which also includes the eosinophil cationic protein (ECP/RNase 3), the angiogenins (ANG/RNase 5), and the epithelial-derived cytotoxin, RNase 7 [25,26,27]. There are currently thirteen human genes recognized as members of the mammalian RNase A family; lower mammalian orders typically have one or more expanded RNase A lineages and many clustered orthologs of these genes [28,29], an interesting feature which will be discussed in greater detail below. The primary sequence of EDN features eight canonically spaced cysteines that generate four specific disulfide bonds that are characteristic of all enzymatically active RNase A-type ribonucleases, as well as histidines 15 and 129 and lysine 38 that generate the catalytic site. The three-dimensional structure of EDN based on crystallographic data is shown in Figure 2 [30].

## 4. Genetics and Diversity of Human EDN and the Mouse Eosinophil-Associated Ribonucleases

Sequences directly orthologous to human EDN and human ECP were readily identified in higher primate genomes, although the degree of inter-species divergence was notably high for otherwise functional coding sequences [31,32]. Interestingly, all primate orthologs maintain the aforementioned cysteines and catalytic elements, although enzymatic activity was not completely conserved throughout [33,34]. Among these findings, Rosenberg and Dyer [33,35] examined the enzymatic activity of EDN orthologs in the genomes of the New World monkeys, the cottontop tamarin (*Saguinus oedipus*) and the Northern owl monkey (*Aotus trivirgatus*). While the EDN orthologs from these primates include the crucial cysteines and catalytic components, and likewise maintain 73% and 71% amino acid sequence similarity to human EDN, respectively, enzymatically, their turnover numbers (k_cat_) were reduced by a factor of about 100. Structure/function analysis indicated that divergence near the C-terminus led to this discrepancy, and that penultimate arginine and/or isoleucine residues were necessary to support the elevated level of enzymatic activity observed in the human ortholog. The constraints promoting divergence in this lineage and among all RNase A-type ribonucleases are a subject of significant and ongoing interest [26,27,36,37].

The human genome includes only one pseudogene of the EDN/RNase 2 lineage [28]. This sequence can be found at position 14q11.2 on human chromosome 14 adjacent to human EDN. While it is of course difficult to comment on the presence or absence of functions unknown, functional pseudogenes are quite rare [38]. In this case, the pseudogene includes components of the EDN open reading frame, which is aborted by aberrant sequence followed by a stop codon shortly after completion of the signal peptide. Trabesinger-Ruef and colleagues [39] presented a consideration of bovine seminal and pancreatic RNases, and postulated that ribonuclease pseudogenes might undergo resurrection to a fully functional state via gene conversion, although whether these findings have specific impact vis à vis EDN and the EDN pseudogene remain uncertain.

Zhang and Rosenberg [40] characterized individual sequence variation at the human EDN locus, and defined nine unique haplotypes. The nucleotide diversity at this locus was within normal limits for a nuclear gene, and one haplotype was represented in >70% of the alleles evaluated. Blom and colleagues [41] built on these findings with the intent of exploring the eosinophil-associated genetic basis of inflammatory bowel disease. In a study that included more than 1000 samples from patients with Crohn’s disease and ulcerative colitis and 300 healthy controls, the authors reported age and gender-dependent differences in association with specific EDN (and ECP)-related haplotypes.

**Figure 3:**
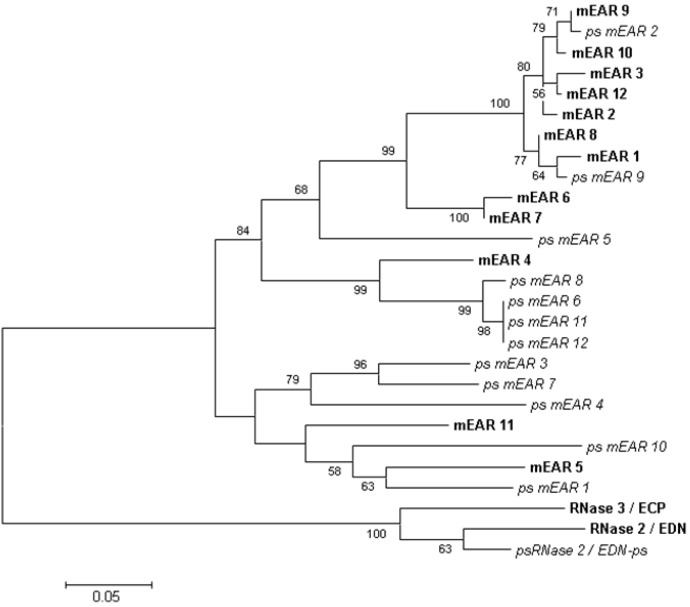
Neighbor-joining tree documenting phylogenetic relationships among the mouse eosinophil-associated ribonuclease genes and pseudogenes. Sequences were aligned using ClustalW; the unrooted tree was created with MEGA 6.0 [47] with bootstrap values (5000 replicates) above 50 as shown.

Larsen and colleagues [42] identified the first rodent orthologs of EDN/ECP in extracts of granules from eosinophils isolated from helminth-infected mice. While mouse eosinophil associated ribonucleases (mEars) 1 and 2 were the primary orthologs identified in eosinophilic leukocytes, the genome of *Mus musculus* encodes a cluster consisting of numerous functional mEar genes and pseudogenes that have diverged via a mechanism characterized as rapid birth-death and gene sorting ([43]; Figure 3). While unusual, this pattern is not unique to the rodent Ears; rapid birth-death has been described as promoting diversity among genes encoding the major-histocompatibility-complex, immunoglobulins, and T cell receptor, all of which are associated with host defense (discussed in [43]). While all clearly orthologous to EDN and ECP, these proteins have on average, only ~50% amino acid sequence similarity to their primate counterparts. Eosinophil-associated gene clusters have been characterized in other rodent species, including hamsters, gerbils, guinea pigs, and rats [43,44]. Interestingly, despite the expansion and sequence diversity, Shamri and colleagues [45,46] have found that eosinophil-active cytokines promote release of mEars from mouse eosinophils via receptor mediated interactions and kinase-dependent mechanism analogous to that utilized by human eosinophils.

The evolutionary constraints driving the rapid evolution of both primate and rodent RNase 2/RNase 3 lineages remain to be fully explored. We and others have hypothesized that duplication and diversification within these lineages has permitted the generation of proteins that can specialize towards promoting unique roles in host defense [31,33,34,43,48].

## 5. EDN and mEars Target Virus Infectivity

**Figure 4:**
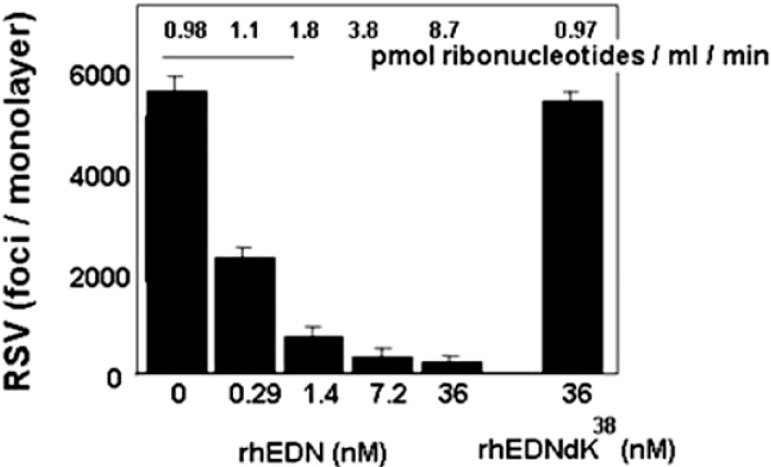
Recombinant human EDN limits the infectivity of the human RSV pathogen for target respiratory epithelial cells *in vitro*. Human EDN displays dose-dependent antiviral activity that is abolished when the protein is rendered catalytically inactive (rhEDNdK^38^). Reprinted from [54] with permission from Oxford University Press.

Asthma exacerbations are commonly triggered by respiratory viruses, which can also have an impact on eosinophilic allergic inflammation [49,50,51]. Activated eosinophils in turn can promote virus clearance, notably in mouse models of allergic airways inflammation [52,53]. Although the mechanisms underlying eosinophil-mediated virus clearance are likely to be multi-faceted, we have shown that EDN can reduce the infectivity of the virus pathogen, human respiratory syncytial virus (hRSV) for target epithelial cells in studies carried out *in vitro* ([54,55]; Figure 4). EDN and also has activity against human immunodeficiency virus in similar tissue culture based assays [56,57]. Likewise, mEar 2 has antiviral activity against pneumonia virus of mice (PVM), a rodent virus related to hRSV; expression of mEars is diminished in mouse lung tissue in response to PVM infection *in vivo* [58]. Furthermore, Gaudrealt and Gosselin [59] found that clearance of influenza *in vivo* correlated with the presence of specific antimicrobial proteins, including the mouse eosinophil-associated RNases. O’Reilly and colleagues [60] found that heterologous expression of mEar1 in mouse lung tissue reduced influenza virus replication and virus-associated leukocyte recruitment. The mechanism(s) via which EDN and/or mEar2 interact with viruses and target cells requires further study. While EDN-mediated antiviral activity against hRSV infection *in vitro* is dependent to a large extent on ribonuclease activity [54], the target molecules and the nature of the interactions remain uncertain. We initially considered the virion as the primary target [54,61]. However, we have since developed a larger vision of the mechanism, and consider the possibility that the ribonuclease may enter the cell during the process of virus infection and inactivate the infected target cell by inducing apoptosis.

## 6. EDN as a Fusion RNase to Target Hepatitis B Virus (HBV)

EDN has been utilized as a component of a genetically engineered fusion protein with hepatitis B virus core protein ((HBVc); [61,62]). Antiviral activity involves expression of the enzymatically-active recombinant fusion protein; the HBVc protein component directs the EDN to nascent virus, most likely targeting an RNA intermediate within the intracellular space. No significant destruction of cellular RNA was reported. While this fusion protein is not a natural antimicrobial RNase, it is interesting to contrast this mechanism with that used by exogenous EDN to inhibit virus infected cells, to the extent that the latter is currently understood.

## 7. Eosinophils Interact with Bacteria and Release EDN

Human eosinophils express a substantial range of pattern recognition receptors (PRRs; reviewed in [63]). Among those PRRs are those that define interactions with exogenous bacteria (*i.e.*, TLR2, TLR4, NOD1 and NOD2), although it is not yet clear how expression of these receptors contributes specifically to eosinophil function in health and disease. Although EDN, among the four granule proteins, does not display broad-spectrum anti-microbial activity [64,65], it is released from eosinophils upon contact with bacterial pathogens. Hosoki and colleagues [66] reported that eosinophils responded with prominent release of EDN when challenged with pathogenic bacteria, such as *Clostridium difficile*, in contrast to a probiotic strain, such as *Bifidobacteria*, which did not induce EDN release. In a subsequent study, eosinophils again released EDN selectively, notably in response to provocation with *Staphylococcus aureus*, but not *Hemophilus* or *Prevotella* species [67]. The way in which eosinophils differentiate between distinct bacterial strains remains to be elucidated.

## 8. EDN and mEar 2 Interact with Dendritic Cells

Yang and colleagues [68,69,70] have characterized several unique ways in which EDN may promote host defense via interaction with leukocytes, specifically with antigen-presenting dendritic cells.

**Figure 5:**
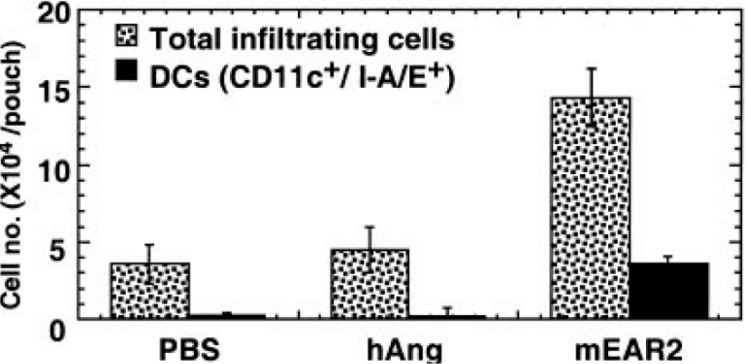
Recombinant mEar2 promotes leukocyte recruitment *in vivo*. Recombinant mEar 2 (but not human angiogenin) elicited recruitment of CD11c^+^ dendritic cells as a fraction of total leukocytes into airpouches. Reprinted from [68] with permission from American Society of Hematology.

First, recombinant EDN and mEar2 were both identified as chemoattractants for CD34^+^progenitor-derived dendritic cells generated in tissue culture; migration was pertussis-toxin sensitive although the receptor has not yet been identified [68]. Furthermore, mEar2 elicited migration of CD11c^+^ DCs (among other cells) into subcutaneous air spaces in experiments performed in BALB/c mice (Figure 5).

EDN, but, interestingly, not mEar2, also induced production of soluble proinflammatory mediators from CD34^+^ progenitor-derived dendritic cells [69]. Among the most prominent responses were the production of IL-6, MCP-3, and IL-12p40, at 182, 97, and 32-fold over background, respectively; similar responses were observed in response to EDN in monocyte-derived DCs.

EDN likewise elicited release of cytokines IL-6 and TNF-alpha from bone marrow-derived mouse dendritic cells, a response directly dependent on the interactions of EDN with the pattern recognition receptor TLR2 [70]; it is not known whether or not enzymatic activity plays a role in this activity as well. As such, EDN has been identified as a member of the alarmins, which are endogenous molecules that interact with pattern recognition receptors, thereby signaling tissue and cell damage [71]. Of interest, Botos and colleagues [72] were the first to document the specific structural similarities between TLRs and ribonuclease inhibitor (RI), the latter a ring-shaped protein with strong affinity for RNase A-type ribonucleases. However, there are no reports of mEar2 or of any mEars promoting cytokine release from dendritic cells or any interactions indicating TLR2-dependence (see also [73]); given the species differences and sequence divergence, this is an important issue worthy of further consideration.

## 9. Mouse Ear 6 and *Schistosoma mansoni* Infection

Of the 12 independent functional mEar genes in the genome of *Mus musculus*, only mEars 1 and 2 are expressed at significant levels at in any currently characterized mouse organ or tissue at homeostasis. However, Nitto and colleagues [74] documented substantial expression of mEar 6 in mouse liver and spleen in response to infection with the helminth pathogen, *Schistosoma mansoni*. Interestingly, this finding was not related to eosinophil infiltration, as mEar 6 expression was not detected in mice that over-express the interleukin-5 transgene. The role of this unique mEar is currently unknown.

## 10. Mouse Ear 11 Is Expressed in Response to IL-4, IL-13, and IL-33 and Is a Macrophage Chemoattractant

While mEar 11 was identified as one of the 12 functional sequences of gene cluster of *Mus musculus*, it is the only mEar-indeed the only RNase A-type ribonuclease-known to be expressed specifically in response to Th2-type cytokines. Cormier and colleagues [75] were first to identify this unique expression pattern, and identified mEar 11 as a product of alveolar macrophages in response to antigen sensitization and challenge or direct administration of IL-4 or IL-13.

**Figure 6:**
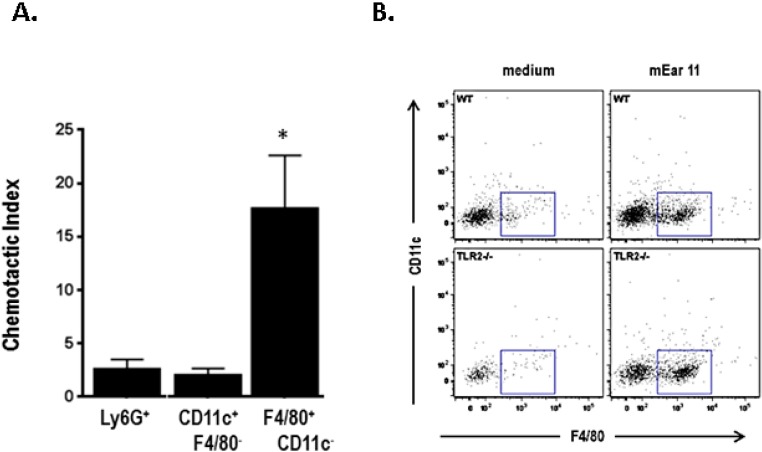
Recombinant *P. pastoris*-derived mEar 11 is chemoattractant for F4/80^+^CD11c^−^ macrophages. (**A**) Chemotactic indices calculated for leukocyte subsets, including Ly6G^+^ (neutrophils), CD11c^+^F4/80^−^ (dendritic cells) and F4/80^+^CD11c^−^ (macrophages); * *p* < 0.05; (**B**) Representative flow plots documenting that F4/80^+^CD11c^−^ splenocytes (within boxes) from TLR2^−/−^ mice migrated in response to mEar 11 to an extent indistinguishable from those from wild-type mice. Reprinted from [73] with permission from American Society for Biochemistry and Molecular Biology.

Yamada and colleagues [73] confirmed this finding, detecting expression of mEar 11 in alveolar macrophages challenged with Th2 cytokines *ex vivo*. Mouse Ear 11 was differentially expressed in somatic tissues at baseline, and its expression was augmented 10–1000-fold in response to administration of IL-33. However, most notable, mEar 11 was identified as a prominent chemoattractant for CD11c^+^F4/80^+^ tissue macrophages, an activity that did not depend on enzymatic activity. Specifically, mEar 11 with a Lys^35^→Arg mutation rendering it enzymatically inactive was equally efficient at attracting mouse macrophages isolated from mouse spleen in experiments performed *ex vivo*. Chemo-attraction likewise did not depend on interactions with macrophage TLR2, as spleen macrophages isolated from mice devoid of this receptor (TLR2^−/−^ mice) responded in a manner that was indistinguishable from their wild-type counterparts. Expression of cell surface markers and cytokine release remains to be examined (Figure 6).

## 11. Future Directions

The RNase A-type ribonucleases, and EDN in particular, remain intriguing subjects for future study. Among the issues that remain to be addressed, we still do not have a clear understanding of enzymatic activity and its role in biological function. Thus, while the antiviral activity of EDN is directly dependent on active ribonuclease activity, these experiments have for the most part been carried out in tissue culture, and need to be explored further in *in vivo* settings. Further research will also focus on how EDN functions as an antiviral mediator, including identification of specific targets (virus *vs.* infected target cells), as well as the potential to harness these observations toward future therapeutic goals. Likewise in need of further clarification are nature, specificity and outcome of the interactions of EDN and potentially the mEars with PRRs. While EDN has been shown to interact with TLR2, future studies may extend these observations to include additional PRRs and likewise may encompass one or more orthologous mEars. Finally, it will be crucial to understand what regulates expression of EDN and mEars in cells other than eosinophils, notably in response to specific classes of proinflammatory mediators. The unique responses of mEar 11 and its expression in macrophages in response to Th2 cytokines and IL-33 may be just the first of a larger series of similar observations. While many questions remain unanswered, the research featured in this review highlights the versatility of EDN and the mEars and the numerous ways in which these RNase A-type ribonucleases may promote host defense.
